# A randomized, double-blind, placebo-controlled 12-week trial of infliximab in patients with juvenile-onset spondyloarthritis

**DOI:** 10.1186/s13075-022-02877-9

**Published:** 2022-08-08

**Authors:** Rubén Burgos-Vargas, Adalberto Loyola-Sanchez, Sofia Ramiro, Arturo Reding-Bernal, Everardo Alvarez-Hernandez, Desirée van der Heijde, Janitzia Vázquez-Mellado

**Affiliations:** 1 Department of Rheumatology, Hospital General de Mexico Dr. Eduardo Liceaga, Mexico City, Mexico; 2 Faculty of Medicine, Universidad Nacional Autonoma de Mexico, Mexico City, Mexico; 3 Division of Physical Medicine and Rehabilitation, Faculty of Medicine and Dentistry, University of Alberta, Edmonton, Canada; 4 Department of Clinical Neurosciences, Alberta Health Services, Edmonton, Canada; 5 Department of Rheumatology, Leiden University Medical Center, Leiden, The Netherlands; 6 Department of Rheumatology, Zuyderland Medical Center, Heerlen, The Netherlands; 7 Research Division, Hospital General de Mexico Dr. Eduardo Liceaga, Mexico City, Mexico

**Keywords:** Spondyloarthritis, Juvenile SpA, Infliximab, Randomized trial, Open-label study, Active joint counts

## Abstract

**Objective:**

To assess the efficacy and safety of infliximab versus placebo in the treatment of patients with juvenile-onset spondyloarthritis (JoSpA).

**Methods:**

Phase III, randomized, double-blind, placebo-controlled trial of 12 weeks that included patients ≤ 18 years old with JoSpA not responding to nonsteroidal anti-inflammatory drugs, sulfasalazine, or methotrexate. Patients were randomly assigned 1:1 to the infusion of infliximab 5mg/kg or placebo; completers entered then an open-label extension (OLE) period of 42 weeks. The primary endpoint was the number of active joints. Secondary outcomes included the assessment of disease activity, tender entheses, spinal mobility, serum C-reactive protein (CRP), the Bath Ankylosing Spondylitis Disease Activity and Functional Index, and the Childhood Health Assessment Questionnaire (CHAQ).

**Results:**

We randomized 12 patients to infliximab and 14 to placebo. No significant differences were found between groups at baseline. At week 12, the mean number of active joints was 1.4 (SD 2.4) in the infliximab group and 4.1 (SD 3.0) in the placebo group (*p* = 0.0002). A repeated-measures mixed model analysis that included all endpoints in the study demonstrated sustained favourable outcomes of infliximab for active joints, tender joints, swollen joints, and tender enthesis counts, as well as for CHAQ and CRP (*p* < 0.01). Adverse events were more frequent in the infliximab group, including infections and infusion reactions, but none of them was serious.

**Conclusion:**

Infliximab is efficacious for patients with JoSpA with an inadequate response to conventional treatment. No serious adverse events with the use of infliximab were observed.

## Key messages

- What is already known about this subject?
  Few randomized clinical trials about the efficacy and safety of tumour necrosis factor-α inhibitors (TNFi) (i.e. etanercept and adalimumab) in patients with juvenile-onset spondyloarthritis (JoSpA) have been published thus far. Regarding infliximab, there is one randomized clinical trial in children with polyarticular-course juvenile idiopathic arthritis, but no JoSpA.
- What does this study add?
  This is the first randomized clinical trial assessing the efficacy and safety of infliximab in patents with JoSpA. At 12 weeks, the mean number of active joints (primary outcome) was significantly lower in the infliximab than in the placebo group. Nearly all secondary measures showed the same result. Infliximab efficacy was sustained during a 42-week open-label phase. Adverse events were seen more often in the infliximab group, but none was serious.
- How might this impact on clinical practice?
  The efficacy of infliximab in this study supports its role in the treatment of children and adolescents with JoSpA.

## Background

Juvenile-onset spondyloarthritis (JoSpA) defines a group of children and adolescents with peripheral enthesitis and arthritis, some of whom are positive for the HLA-B27 gene [1]. At onset, they rarely have axial skeletal involvement [2, 3]. Five to 10 years later, around 75% of them may have involvement of the spine and sacroiliac joints [4–6] and fulfil the modified New York (mNY) criteria for ankylosing spondylitis [7].

In contrast to JoSpA, most patients with adult-onset SpA present with inflammatory back pain and less frequently peripheral arthritis [8]. In the past, the recognition of AS and radiographic sacroiliitis could take up to 10 years [9]. Today, with the use of magnetic resonance imaging (MRI), the recognition of sacroiliitis occurs at an earlier stage [10, 11], preventing symptoms and stop disease progression [11].

The treatment of JoSpA resembles that of adult-onset SpA and some categories of juvenile idiopathic arthritis (JIA) [12]. Yet, there is no evidence that conventional synthetic disease-modifying anti-rheumatic drugs (csDMARDs) might improve JoSpA symptoms [13]. Until now, the use of biological DMARDs (bDMARDs), specifically tumour necrosis factor-α inhibitors (TNFi), has been a major advance in JoSpA treatment. Nevertheless, only four randomized clinical trials, two each on etanercept [14, 15] and adalimumab [16, 17], have been published thus far. Infliximab, a monoclonal TNFi, has only been investigated in children with polyarticular-course JIA [18, 19] and not in patients with JoSpA.

Therefore, we have assessed the efficacy and safety of infliximab versus placebo in children and adolescents with active JoSpA. Long-term efficacy and safety were assessed in an open-label phase of the trial.

## Patients and methods

### Design

**Fig. 1:**
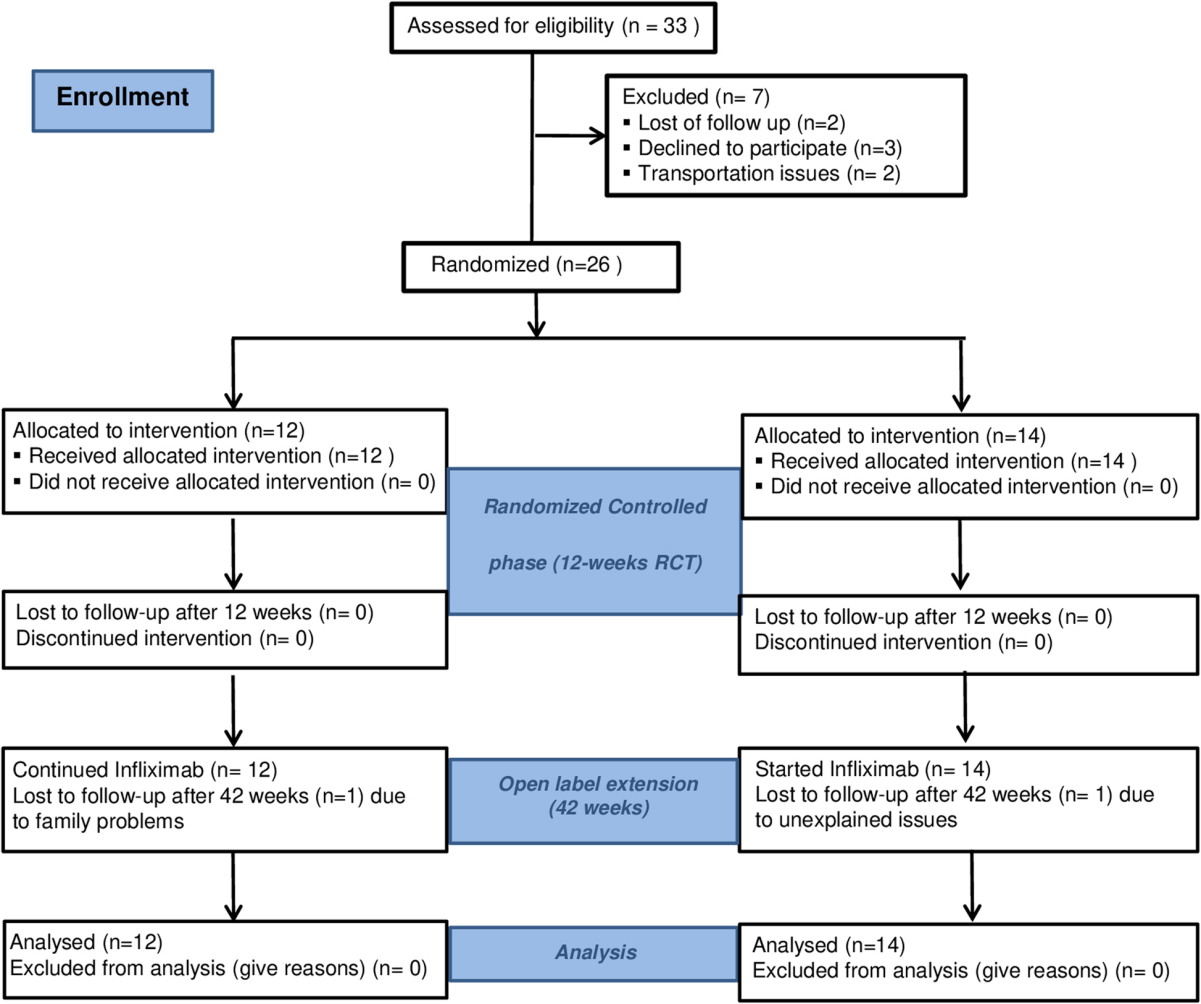
CONSORT flowchart of the infliximab in juvenile-onset SpA trial

This was a phase III, randomized, double-blind, placebo-controlled, 12-week study followed by a 42-week open-label extension (Fig. 1).

### Ethical considerations

The study was conducted following the Declaration of Helsinki and International Conference on Harmonization Good Clinical Practice including respect for individual’s beneficence, justice, and autonomy. The Research and Ethics Review Board of the Hospital General de Mexico Dr. Eduardo Liceaga approved the conduction of this trial (Hospital General de Mexico Research Division Registry: HGM/DIC/02/404-B/02/036). Patients, parents, or legal guardians and two witnesses were informed about the study and if accepted to participate signed an informed consent form. The protocol was registered in ClinicalTrials.gov (identifier: NCT00591201).

### Participants

Eligible patients were children and adolescents with active JoSpA who fulfilled the children’s validation of the European Spondyloarthropathy Study Group (ESSG) classification criteria for SpA [20, 21] and were mainly recruited from the paediatric rheumatology clinics at the Hospital General de México Dr Eduardo Liceaga. Some participants were referred from private rheumatologist offices and the Shriners Hospital for children in Mexico City. Recruitment took place from June 2002 to June 2007. Inclusion criteria were age ≤ 16 years at the onset of symptoms and ≤18 years at screening. Disease activity required four criteria: (1) ≥ 3 active joints (see below); (2) ≥ 3 tender peripheral entheses; (3) ≥ 4 points of pain intensity on a 10-point numerical rating scale (NRS) (0 = no pain, 10 = the worst possible pain); and (4) no clinical improvement or intolerance to the administration of ≥ 2 nonsteroidal anti-inflammatory drugs (NSAIDs), csDMARDs, and systemic glucocorticoids.

Key exclusion criteria were active extra-musculoskeletal manifestations, such as psoriasis, anterior uveitis, and Crohn’s disease; comorbidities or medications interfering with the course of the trial; suspected or confirmed diagnosis of tuberculosis or other chronic infections; lymphoma or any other neoplasia; previous therapy with TNFi; lack of vaccinations, particularly Bacillus Calmette-Guerin (BCG); a positive skin test (>5 mm) following the subcutaneous injection of the purified protein derivative (PPD); and for sexually active boys and girls, use of less than 2 contraceptive measures.

Patients were allowed to continue on NSAIDs, oral prednisone or its equivalent (<10 mg/day), SSZ (≤ 50 mg/kg/day), and MTX (≤ 15 mg/m^2^/body surface area) as long as there were no changes in the dosages during the study.

### Procedures

During the double-blind phase, patients were randomly assigned 1:1 and allocated to infliximab 5 mg/kg or placebo infusions at weeks 0, 2, 6, and 12 according to a computer-generated randomization list restricted by blocks of four. Patients, parents, and investigators were blinded to allocation. Patients who finished the double-blind 12-week period were invited to participate in the open-label extension. Patients on infliximab continued with infusions every 6 weeks. Patients on placebo started receiving infliximab on week 12 and then every 6 weeks. Patients with serious adverse events or worsening of their disease in the double-blind phase were allowed to change to the open-label phase or withdraw from the trial.

### Outcomes

The primary outcome was the number of active joints (0 to 68), defined by the presence of swelling or range of motion limitation and pain and/or tenderness [22]. Secondary outcomes included joint counts for tenderness (0–72), swelling (0–68), and reduced mobility (0–66); number of tender entheses (0–55); serum high-sensitive C-reactive protein (hsCRP) in milligrammes per decilitre; and the following spinal measurements: the modified Schober’s test (cm), lateral spinal flexion (cm), chest expansion (cm), and neck and hip rotation (degrees).

We obtained the physician’s global assessment (PGA) of disease activity, parent’s rating of participants’ pain and global well-being, all in 10-point numeric rating scales (NRS), and we calculated the Bath Ankylosing Spondylitis Disease Activity Index (BASDAI) [23], the Bath Ankylosing Spondylitis Functional Index (BASFI) [23], and the Childhood Health Assessment Questionnaire (CHAQ) [24] using cross-culturally adapted instruments. Even though children above 7 years old had the capacity to answer most questionnaires on their own, for the purpose of this study, we only included the responses given by parents or legal guardians.

We calculated the percentage of patients that achieved the American College of Rheumatology (ACR) Paediatric (Pedi) 30 response [25]. This response was defined as three of any of six variables: (1) PGA of disease activity, (2) parent/patient global assessment of overall well-being, (3) functional ability, (4) number of joints with active arthritis, (5) number of joints with limited range of motion, and (6) hsCRP improving 30% or more with no more than one of the remaining variables worsening more than 30%. We also estimated the ACR-Pedi 50, 70, 90, and 100 responses.

The Assessment in Ankylosing Spondylitis (ASAS) 20 [26], ASAS 40 [27], and ASAS 5/6 [27] improvement and ASAS partial remission criteria [26] were calculated according to four domains: (1) patient global assessment of disease activity, (2) spinal pain, (3) function (mean BASFI), and (4) inflammation (mean BASDAI questions 5 and 6). ASAS 5/6 also consider the hsCRP and lateral spinal flexion variables. To fulfil ASAS 20 criteria, an improvement of ≥ 20% and ≥ 1 unit in 3 or 4 domains plus no worsening ≥ 20% or ≥ 1 unit in the remaining domain was necessary. The ASAS 40 criteria required improvement of ≥ 40% and ≥ 2 units in 3 or 4 domains plus no worsening in the remaining domain. The ASAS 5/6 criteria required an improvement ≥ 20% in at least 5 domains and the ASAS partial remission criteria required each of the 4 domains to be ≤ 2 units.

### Safety evaluation

Adverse events (AEs) were collected from the first infusion of treatments onwards, including infections such as tuberculosis, malignancies, and infusion reactions. The definition of AEs followed those of the Medical Dictionary for Regulatory Activities (MedDRA). AEs were expressed as number of patients and percentages. Serious adverse events (SAEs) were also monitored (i.e. death, life-threatening condition, hospital admission, hospital stay extension, and disability).

### Statistical analysis

The primary analysis followed an intention-to-treat (ITT) strategy and included all participants who received at least one infusion of Infliximab. To assess the in-between group differences for number of active joints and continuous secondary outcomes, we used the analysis of covariance (ANCOVA) adjusted for baseline scores [28]. For nominal outcomes, we utilized Fisher’s exact test to assess the differences between groups at week 12. In addition, we performed the repeated-measures mixed model analyses for continuous outcomes. These analyses assessed group and time interactions in both the double-blind and open-label phases of the study. To calculate the sample size, we used the independent means difference method considering an intervention effect size of 1.1 (Cohen’s *d*) on the number of active joints, based on expert opinion since no literature was available when this study was designed. We sought for a statistical power of 80% with a confidence level of 95% in a two-tailed hypothesis. Fourteen participants were estimated to be needed for each group. All analyses were performed using STATA version 16.

## Results

Twenty-six patients that fulfilled the ESSG criteria were randomized, 14 to placebo and 12 to infliximab (see Fig. 1). All patients completed the randomized controlled phase, and none was switched early to the open phase. Two patients withdrew their consent at weeks 24 and 30 because of family problems at home and unknown reasons, respectively. There were no significant demographic or clinical differences between the groups at baseline (see Table 1). Two patients in each group fulfilled the mNY for AS or r-axSpA, whereas all patients fulfilled the International League of Associations for Rheumatology (ILAR) ERA classification criteria

**Table 1:** Baseline demographics and disease characteristics

	Placebo (***n*** = 14)	Infliximab (***n*** = 12)	***p***^c^
**Demographics**			
Males, no. (%)	13 (92.9)	12 (100.0)	1.000
Age, years mean (SD)	14.5 (2.7)	15.0 (1.7)	0.587
Weight, kg mean (SD)	55.2 (19.5)	52.7 (14.5)	0.718
Body mass index, kg/m^2^ mean (SD)	21.4 (5.1)	19.7 (3.3)	0.339
Radiographic sacroiliitis ^a^, no. (%)	2 (14.3)	2 (6.7)	0.763
**Disease characteristics**			
Disease duration, years mean (SD)	6.9 (3.5)	6.4 (2.7)	0.689
HLA-B27, no. (%)	13 (92.9)	10 (90.9)	0.859
Family history of SpA^b^, no. (%)	2 (14.3)	1 (8.3)	0.642

*no*. number, *SD* standard deviation

^a^Radiographic sacroiliitis if at least graded 2 or 3 bilateral or 3 unilateral

^b^Family history of SpA refers to three cases with axSpA, one to nrSpA, and another to rSpA (AS)

^c^Probability values of true differences, utilizing parametric *t*-tests for continuous variables and non-parametric chi-squared tests for nominal variables

### Efficacy

**Fig. 2:**
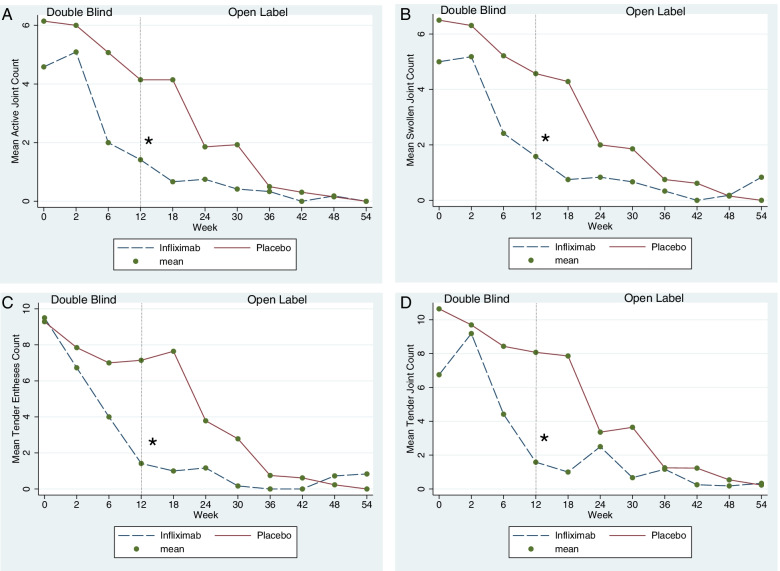
Mean active joint counts, swollen joint counts, tender joint counts, and tender enthesis counts registered during the entire duration of the study (RCT + OLE phases) by treatment group according to randomization. **A** Mean active joint count (primary outcome). **B**–**D** Mean number of swollen joints, tender joints, and tender enthesis, respectively. All comparisons showed a significant difference between infliximab and placebo by week 12. In the open-label extension, in which all patients received infliximab, the mean of each outcome showed a sustained response to infliximab

**Fig. 3:**
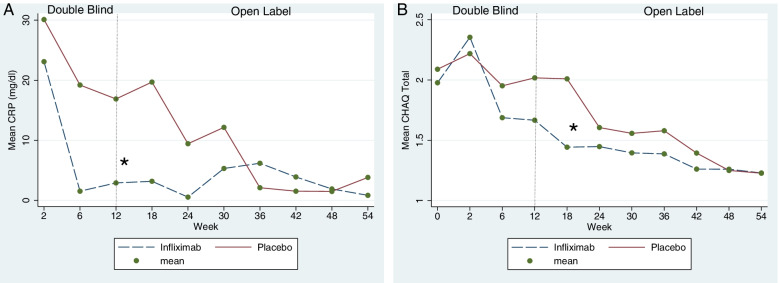
Mean level of high-sensitive C-reactive protein (hsCRP) and Childhood Health Assessment Questionnaire score (CHAQ) registered during the entire duration of the study (RCT + OLE phases) by treatment group according to randomization. **A** Mean hsCRP serum levels in milligrammes per decilitre. **B** Mean CHAQ scores. Lines showed a significant and sustained positive effect of infliximab over time

At the end of the randomized controlled phase of the trial, the number of active joints was lower in the infliximab [1.4 (SD 2.4)] than in the placebo [4.1 (SD 3.0)] group (*p* = 0.0002) (Fig. 2A). Similar results were obtained for the mean number of swollen (Fig. 2B) and tender joints (Fig. 2C), entheses (Fig. 2D), and hsCRP (Fig. 3A). All these differences were statistically significant (*p* < 0.01) (see Table 2).

**Table 2:** Between-group differences in primary and secondary continuous outcomes at baseline and at the end of the RCT phase

	Placebo		Infliximab		
	Baseline	Week 12	Baseline	Week 12	p*
Active joints, no.	6.1 (3.7)	4.1 (3.0)	4.5 (1.7)	1.4 (2.4)	0.0002
Tender joints, no.	10.6 (6.8)	7.8 (7.9)	6.7 (3.1)	1.0 (2.0)	0.0001
Swollen joints, no.	6.5 (3.6)	4.5 (3.0)	5.0 (2.0)	1.5 (2.4)	0.0003
Tender entheses, no.	9.2 (4.8)	7.1 (5.9)	9.5 (9.7)	1.4 (2.3)	0.004
CHAQ score, 0–3	2.0 (0.5)	2.0 (0.4)	1.9 (0.5)	1.6 (0.8)	0.1
BASDAI score, 0–10	6.1 (1.9)	5.5 (1.8)	5.5 (2.5)	3.4 (2.3)	0.07
BASFI score, 0–10	5.4 (2.6)	4.9 (2.0)	5.5 (2.8)	3.0 (2.8)	0.12
hsCRP level, mg/dl	30.1 (23.4)	19.7 (17.3)	23.1 (9.5)	3.1 (5.0)	0.003
Modified Schober’s, cm	4.5 (1.1)	4.6 (1.3)	4.5 (1.5)	4.7 (1.0)	0.84
Lateral flexion, cm	17.6 (7.0)	16.7 (5.4)	25.4 (14.6)	18.1 (7.1)	0.81
Chest expansion, cm	4.5 (1.5)	4.8 (1.4)	4.4 (1.3)	4.7 (1.5)	0.94
Hip rotation, cm	39.9 (8.9)	42.3 (11.8)	50 (16.1)	47.4 (9.9)	0.92
Physician assessment of disease activity, 10-cm NRS	6.7 (1.6)	5.1 (2.8)	6.4 (1.0)	1.3 (2.1)	0.0006
Physician assessment of health status, 10-cm NRS	3.7 (2.1)	5.0 (2.7)	4.1 (1.3)	7.6 (2.0)	0.01
Parent/patient assessment of well-being, 10-cm NRS	6.4 (1.3)	5.3 (2.6)	3.8 (1.9)	2.3 (2.2)	0.39
Pain score, 0–10 NRS	7.5 (1.8)	5.8 (2.7)	7.2 (1.9)	2.4 (1.9)	0.003

Values represent the mean (SD)

*BASDAI* Bath Ankylosing Spondylitis Disease Activity Index, *BASFI* Bath Ankylosing Spondylitis Functional Index, *CHAQ* Childhood Health Assessment Questionnaire, *hsCRP* high-sensitive C-reactive protein

*p-values reflect the comparison of the outcomes at week 12 and obtained with ANCOVA analysis adjusting for baseline values

The results from the repeated-measures mixed model analyses showed that the evolution of the continuous outcome measures over time was different between groups and clearly favoured infliximab (Figs. 2 and 3). This was demonstrated by significant interactions between time and treatment group in these models (active joint count *p* = 0.0001; tender joint count *p* < 0.001; swollen joint count *p* < 0.001; tender enthesis count *p* < 0.001; hsCRP levels *p* < 0.001; and the CHAQ scores *p* = 0.004).

Differences between groups in CHAQ, BASDAI, and BASFI scores and Schober’s, lateral flexion, chest expansion, hip rotation, and parent assessment of well-being were not significant at week 12. Physician’s assessment of disease activity, health status, and parents’ reports on pain yielded significant differences favouring the infliximab group at the end of the randomized clinical trial (RCT) phase (see Table 2).

**Fig. 4:**
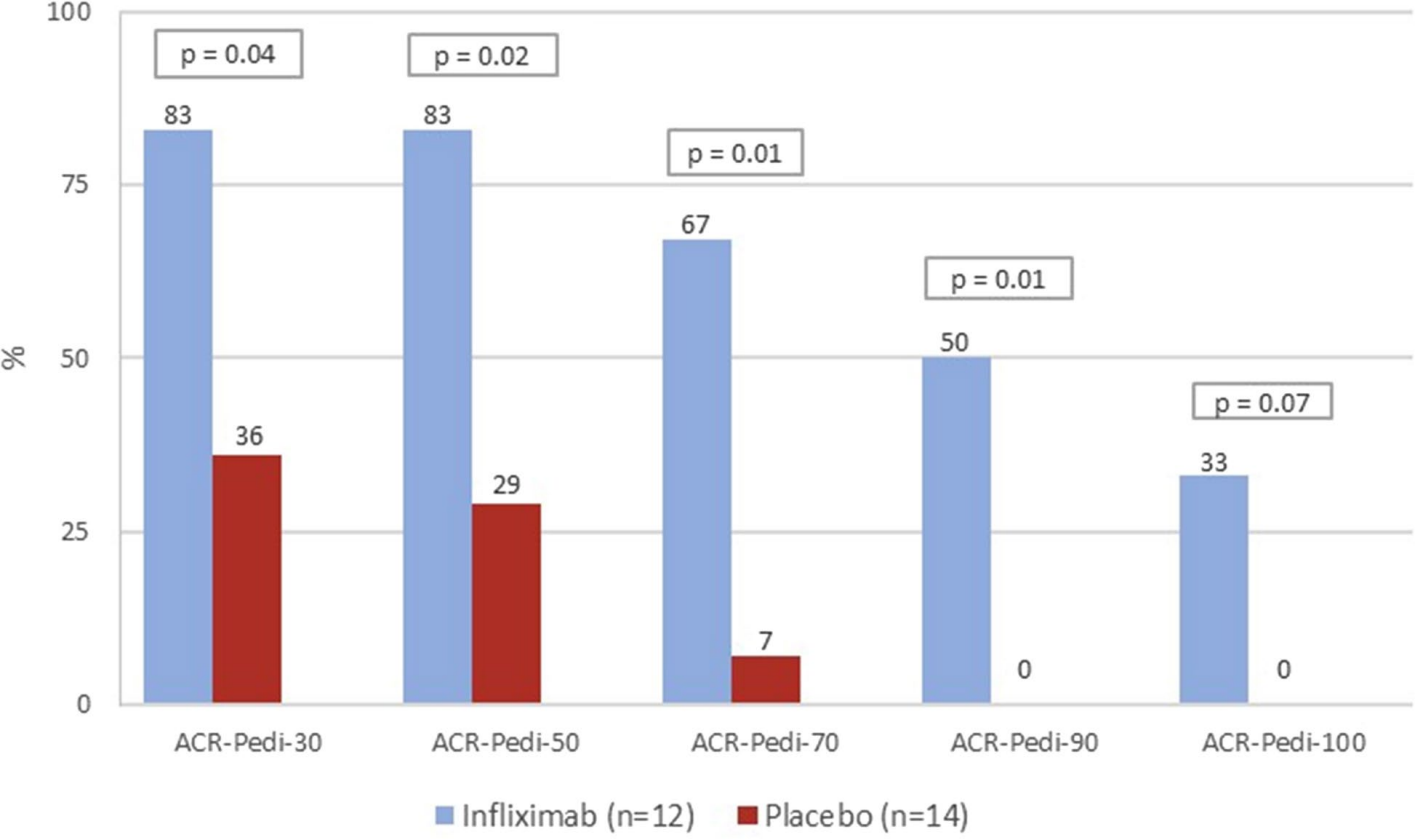
Percentage of patients reaching the American College of Rheumatology (ACR) Paediatric 30 (Pedi 30), 50, 70, 90, and 100 response criteria per treatment group at week 12 (end RCT phase)

The proportions of patients achieving the ACR-Pedi 30, 50, 70, and 90 responses were significantly higher in the infliximab group (Fig. 4). Despite that 33% of the patients in the infliximab group showed an ACR-Pedi 100 response vs none in the placebo group (Fig. 4), the small number or patients did not allow to achieve statistical significance.

**Fig. 5:**
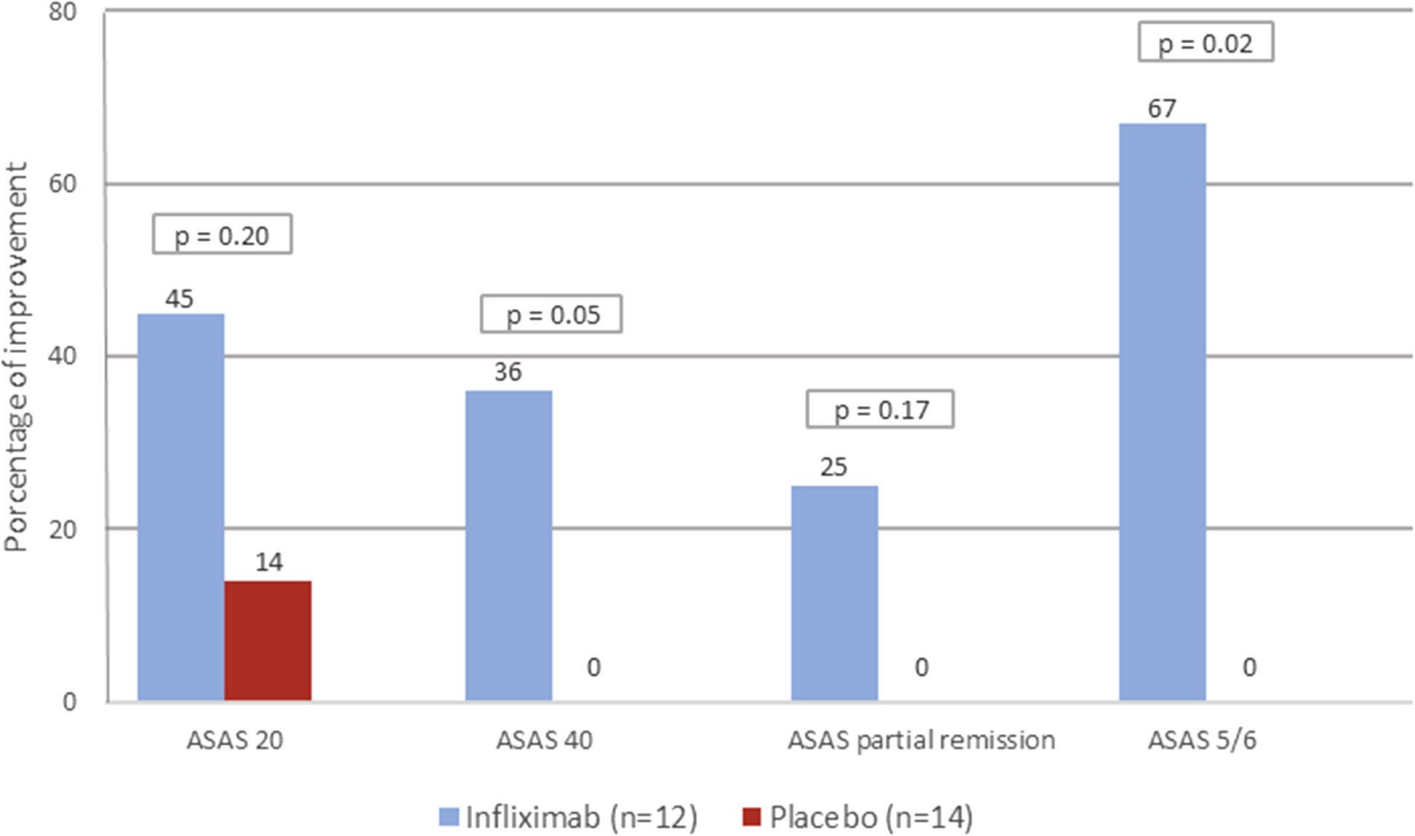
Percentage of patients reaching the Assessment of Spondyloarthritis international Society (ASAS) 20, 40, partial remission, and 5/6 response criteria per treatment group at week 12 (end RCT phase)

More patients in the infliximab group achieved ASAS40 and ASAS5/6 responses (Fig. 5). However, differences between groups on ASAS20 (infliximab = 45% vs placebo = 14%) and ASAS partial remission (infliximab = 25% vs placebo = 0%) responses were not statistically significant, despite a clear higher number of patients achieving them in the infliximab group (Fig. 5).

### Adverse events

The overall number of patients with any AE was nine (75%) in the infliximab and eight (57%) in the placebo groups (Table 3). Infections were more frequent in patients who received infliximab (41% vs 28%) and participants presented with different types of infections during the study, which are described in Table 3. Infusion reactions were more common in the infliximab group (41%) than in the placebo group (7%), but none of them was considered serious (i.e. fever, headache, and dizziness). Two patients on infliximab developed psoriatic plaques and nocturnal back pain after 4 weeks, and these were considered paradoxical adverse events. Importantly, none of the patients developed SAEs during the study.

**Table 3:** Adverse events (AEs) during the complete duration of the study (RCT + OLE)

		Infliximab (*n* = 12)	Placebo (*n* = 14)
Patients with			
	Any adverse event (%)	9 (75)	8 (57.1)
	Any infection (%)	5 (41.6)	4 (28.5)
	Infusion reactions (%)	5 (41.6)	1 (7.4)
	Serious AEs^a^	0 (0.0)	0 (0.0)
Infection related			
	Varicella	1 (8.3)	1 (7.4)
	Pharyngitis	3 (25.0)	4 (28.5)
	Upper tract respiratory infections, including flu	5 (41.6)	5 (35.7)
	Diarrhoea	2 (16.6)	2 (14.3)
Infusion related			
	Fever	3 (25.0)	2 (14.3)
	Headache	1 (8.3)	0 (0.0)
	Dizziness	1 (8.3)	0 (0.0)
Paradoxical AEs			
	Psoriasis	1 (8.3)	0 (0.0)
	Back pain	1 (8.3)	0 (0.0)

*RCT* randomized controlled trial phase, *OLE* open-label extension phase

^a^Serious adverse events monitored included, death, life-threatening condition, hospital admission, hospital stay extension and disability

## Discussion

This study demonstrated that infliximab is effective and safe in the treatment of JoSpA. The primary endpoint, namely the number of active joints, was significantly lower in patients on infliximab compared to placebo. Moreover, most secondary outcomes, including composite scores and response criteria, showed improvements in favour of infliximab. The repeated mixed model analyses showed sustained efficacy of infliximab on the primary and most secondary outcomes during the open-label phase. AEs were mild and mostly related to infections and infusion reactions.

Although the prevalence of JoSpA in Paediatric Rheumatology clinics is relatively low, the disease seems more active and severe than other JIA categories and adult-onset SpA [29, 30]. The risk of developing AS within 10 years from onset is higher in HLA-B27 boys who are ≥ 8 years old and present with foot arthritis, enthesitis along with hip, sacroiliac, and spinal involvement at onset [31–34]. Before bDMARD use, remission occurred in 20% of patients with JoSpA. At 15-year follow-up, the disease remained active in 50% of the cases. Disease activity at baseline predicted functional impairment by 10 years in 60% of patients [33, 35, 36]. Moreover, patients with JoSpA score higher in CHAQ [32, 37] and bodily pain [38, 39] than other JIA categories.

Up to date, the efficacy of bDMARDs in children with JoSpA has not been established. However, their effect in polyarticular JIA and adults with axSpA supports their use in children and adolescents with JoSpA and enthesitis-related arthritis (ERA). JIA categories differ from each other regarding prevalence, clinical features, outcome measures, and management. Considering ethical constraints in the conduction of clinical trials in children, the use of a controlled withdrawal design has become the standard for clinical trials in JIA, even though the appropriateness of this standard has been questioned [40–42]. Therefore, most of the clinical trials on the use of TNFi in JoSpA utilized a controlled withdrawal design and only one has utilized a standard RCT design [15], which we implemented in the present study.

Two open-label studies on the efficacy of bDMARDS in the JoSpA population have been published. One open-label study on etanercept for 24 weeks clearly showed efficacy in an effect of the TNFi in preventing disease flares [43]. Similarly, the open-label CLinical Study in Paediatric Patients of Etanercept for treatment of ERA, PsA, and extended oligoarthritis (CLIPPER) [14, 15, 44] showed efficacy of the biologic to achieve the ACR-Pedi 30 response at 12 weeks. This study utilized two historical groups [14, 17].

Two RCTs evaluated the efficacy of adalimumab in the JoSpA [16, 17]. Conducting placebo-controlled RCTs in the JoSpA population has several advantages. One of the mentioned adalimumab RCTs included 32 patients with juvenile-onset ankylosing spondylitis (JoAS) and found no statistically significant differences between adalimumab and placebo in the achievement of ASAS 40 response as their primary endpoint; however, it showed significant effects in other outcomes [17]. The other RCT on adalimumab included 46 patients with ERA and found a clear positive effect of the TNFi on the percentual change from baseline in the number of active joints in comparison to placebo [16]. Utilizing the “percentual change from baseline” to attribute intervention causality in RCTs has been shown ineffective due to this strategy’s high sensitivity to changes in variance, reducing its power to detect true differences [28]. Additionally, in our study, we opted for ANCOVA adjusting for baseline outcomes for the between-group comparison of means at the end of the RCT as a more appropriate manner to assess between-group differences.

Even though we selected the same RCT design as the previous adalimumab studies, we implemented more stringent criteria to select patients with higher disease activity. We believe that our disease activity parameters are more in line with what is currently used to identify children who require bDMARDs. Inclusion criteria of other studies have been more permissive including a wider range of disease activity, which could go from two active joints in CLIPPER [14] to a combination of sacroiliac, spinal, oligoarthritis, and imaging studies in the JoAS trial [17].

With respect to our primary outcome selection, the ACR-Pedi 30 response criteria have been the primary endpoint of RCT withdrawal trials, which included the ACR criteria for flare [43]. The adalimumab trials included ASAS40 response as a primary outcome [16, 17]. We selected the number of active joints as our primary outcome, due to its clinical implications, which strongly indicates the disease severity in this population. In addition, our results demonstrate that this outcome is sensitive to change, and clinically important differences can be found even in small samples.

There were no SAEs reported in this study. However, more patients on infliximab had infectious and infusion-related reactions compared with placebo. In contrast, a trial of infliximab in 122 patients with polyarticular-course juvenile rheumatoid arthritis randomized to 3 mg/kg or 6 mg/kg yielded AEs in 95% of the patients [18, 19], including serious AEs in 32% of infliximab users. The Pharmachild (*n* = 2022; all licensed bDMARDs) and Biker (*n* = 1697; all licensed bDMARDs) registries barely mention the use of infliximab suggesting low utilization of this TNFi in the paediatric population in some countries [45–49]. Interestingly, few AEs have been reported with the use of high doses of infliximab in refractory cases of JIA extra-musculoskeletal manifestations such as uveitis [50, 51].

The limitations of our study could be related with the potential comparability with future studies, particularly the fact that we did not use composite measures as our primary outcome. Some of the newest composite measures, such as the AS Disease Activity index [52, 53], the juvenile SpA Disease Activity index [54], and the Juvenile Arthritis Disease Activity Index (JADAS) [55], were not available when we designed the study and have since been developed to improve the assessment of patients with JoSpA and can be used in future clinical trials. Another limitation was related with the sample size calculation, as by the time we planned the study there were no clinical trials on the use of TNFi for JoSpA in the literature. Therefore, the power of our study was determined by experts’ opinion on the significant difference expected among groups. The fact that our sample size was enough to detect significant differences between groups on our primary outcome makes this limitation less relevant for the conclusions that emerge from our findings. Finally, we did not utilize MRI studies to define our population and monitor structural changes after the intervention. This could have resulted in the non-identification of potential participants who were in early stages of disease and could have responded better to this intervention.

## Conclusions

In summary, the infusion of infliximab at a loading dose of 5 mg/kg for 12 weeks and then every 6 weeks up to 54 weeks was effective to reach a lower number of active joints, tender joints, swollen joints, tender entheses, hsCRP levels, and better levels of physical function in children and adolescents with active JoSpA. Consequently, we conclude that infliximab is efficacious and is a good treatment alternative for JoSpA.

## Data Availability

The anonymized database utilized in the analysis of this manuscript is available upon request.

## Abbreviations

ACR: American College of Rheumatology
AEs: Adverse events
ANCOVA: Analysis of covariance
ASAS: Assessment in Ankylosing Spondylitis
BASDAI: Bath Ankylosing Spondylitis Disease Activity and Functional Index
BASDAI: Bath Ankylosing Spondylitis Disease Activity Index
BASFI: Bath Ankylosing Spondylitis Functional Index
BCG: Bacillus Calmette-Guerin
CHAQ: Childhood Health Assessment Questionnaire
CLIPPER: CLinical Study in Paediatric Patients of Etanercept for treatment of ERA, PsA, and extended oligoarthritis
CRP: Serum C-reactive protein
csDMARDs: Conventional synthetic disease-modifying anti-rheumatic drugs
ERA: Enthesitis-related arthritis
ESSG: European Spondyloarthropathy Study Group
hsCRP: High-sensitive C-reactive protein
ILAR: International League of Associations for Rheumatology
ITT: Intention to treat
JADAS: Juvenile Arthritis Disease Activity Index
JIA: Juvenile idiopathic arthritis
JoAS: Juvenile-onset ankylosing spondylitis
JoSpA: Juvenile-onset spondyloarthritis
JoSpA/ERA: Juvenile-onset spondyloarthritis and enthesitis-related arthritis
MedDRA: Medical Dictionary for Regulatory Activities
mNY AS: Modified New York criteria for Ankylosing Spondylitis
MRI: Magnetic resonance imaging
MTX: Methotrexate
NRS: Numerical rating scale
OLE: Open-label extension phase
Pedi: Paediatric
PGA: Physician’s global assessment
PPD: Purified protein derivative
r-axSpA: Radiographic axial spondyloarthritis
SpA: Spondyloarthritis
SSZ: Sulfasalazine
TNFi: Tumour necrosis factor-α inhibitors
